# *MLH1* Methylation Testing as an Integral Component of Universal Endometrial Cancer Screening—A Critical Appraisal

**DOI:** 10.3390/cancers15215188

**Published:** 2023-10-28

**Authors:** Anna Plotkin, Ekaterina Olkhov-Mitsel, Sharon Nofech-Mozes

**Affiliations:** 1 Department of Laboratory Medicine and Molecular Diagnostics, Division of Anatomic Pathology, Precision Diagnostics and Therapeutics Program, Sunnybrook Health Sciences Centre, Toronto, ON M4N 3M5, Canada; 2 Laboratory Medicine and Pathobiology, University of Toronto, Toronto, ON M5S 1A8, Canada

**Keywords:** endometrial cancer, lynch syndrome, mismatch repair deficiency testing, *MLH1* promoter methylation analysis, gynecologic pathology, immunohistochemistry, microsatellite instability

## Abstract

**Simple Summary:**

Often in endometrial cancer (EC), the mismatch repair (MMR) system, which helps fix DNA mistakes, goes haywire because of *MLH1* promoter hypermethylation (*MLH1*-PHM). We set out to find out how many ECs have *MLH1*-PHM, understand the impact of reflex *MLH1*-PHM testing and evaluate the associated costs within the publicly funded Canadian healthcare system. We looked at 2504 EC samples and found that in 534 of them (21.4%), the MMR system was deficient due to dual MLH1/PMS2-deficiency. Out of the 418 cases with available data, 404 (96.7%) had *MLH1*-PHM, while only 14 (3.3%) didn’t. Reflex *MLH1*-PHM tests cost CAD 231.90 per case, amounting to CAD 123,834.60 for 534 cases, with 30 tests needed per additional candidate for *MLH1* germline analysis (CAD 6,957.00 per candidate). This raises a provocative question: can we assume that the majority of the MLH1-deficient ECs are due to PHM and forgo further testing in healthcare systems with finite resources?

**Abstract:**

MLH1/PMS2 loss due to *MLH1* promoter hypermethylation (*MLH1*-PHM) is the most common cause of mismatch repair (MMR) deficiency in endometrial cancer (EC). This study aimed to determine the proportion of *MLH1*-deficient EC with PHM, assess the impact of the reflex *MLH1*-PHM testing strategy, and evaluate the associated costs within the publicly funded Canadian healthcare system. In a cohort of 2504 EC samples, 534 (21.4%) exhibited dual MLH1/PMS2 loss, prompting *MLH1*-PHM testing. Among 418 cases with available testing results, 404 (96.7%) were *MLH1*-hypermethylated, while 14 (3.3%) were non-methylated. The incidence of *MLH1* non-methylated cases in our cohort was 14/2504 (0.56%) of all ECs, underscoring the prevalence of hypermethylation-driven MLH1/PMS2 loss in ECs universally screened for MMR deficiency. Reflex *MLH1*-PHM testing incurs substantial costs and resource utilization. Assay cost is CAD 231.90 per case, amounting to CAD 123,834.60 for 534 cases, with 30 tests needed per additional candidate for *MLH1* germline analysis (CAD 6957.00 per candidate). This raises a provocative question: can we assume that the majority of the MLH1-deficient ECs are due to PHM and forgo further testing in healthcare systems with finite resources? It is imperative to assess resource utilization efficiency and explore optimized approaches that encompass clinical correlation, family history and judicious utilization of methylation testing to ensure it is provided only to those who stand to benefit from it.

## 1. Introduction

Endometrial cancer (EC) is the second most prevalent gynecologic cancer worldwide, with an annual increase in morbidity of 0.6% and mortality of 2% [1,2]. While the majority of cases are sporadic, a subset of women, estimated at 2–5%, harbor an inherited DNA mismatch repair (MMR) mutation, resulting in Lynch syndrome (LS)-associated EC [3,4]. Loss-of-function pathogenic variants in one of the four MMR genes (*MLH1*, *MSH2*, *MSH6*, or *PMS2*), and occasionally the related gene *EPCAM*, result in the accumulation of single-nucleotide variants, insertions, and deletions, particularly in microsatellites, the repetitive regions of DNA [5]. Tumors with this phenotype are termed microsatellite unstable (MSI) or MMR-deficient (MMR-d).

As classification of EC transitions from histologic-based to molecular-based risk stratification, determining MMR/MSI status has a role beyond the identification of LS [6]. Accordingly, it has a fundamental role in the molecular classification of EC tumors, as defined by The Cancer Genome Atlas (TCGA), including *POLE* ultramutated, MMR-d, copy number high (p53 abnormal) and copy number low (no specific molecular profile) [7]. Molecular EC classification improves disease prognostication and provides predictive information that guides personalized therapy [8,9]. *POLE* ultramutated ECs have a favorable prognosis and may not require adjuvant treatment, while MMR-d and copy number low ECs have an intermediate prognosis and copy number high ECs experience the least favorable prognosis [10]. Notably, MMR-d and copy number low tumors express high levels of progesterone receptors, suggesting potential responsiveness to fertility-preserving hormonal therapy, which is particularly important for the quality of life and future prospects of young EC patients [11,12]. Recent analyses of prospective clinical trials showed no significant benefit from standard chemotherapy and persistent risk of recurrence following brachytherapy in MMR-d EC [8]. Moreover, MMR-d ECs have been shown to derive benefit from the addition of immunotherapies [13,14]. Current evidence shows no difference in risk stratification or treatment allocation between MMR-d caused by LS or sporadic promoter methylation, although such differences may emerge in the future when analyses of ongoing clinical trials become available [15].

Due to its dual purpose in LS screening and molecular classification, universal screening of ECs for MMR-d is recommended by NCCN guidelines and has become the standard-of-care in many jurisdictions, including Canada [16,17,18,19]. This is commonly achieved through the utilization of immunohistochemistry (IHC) for the detection of MMR protein status [18,20]. Notably, while only a small fraction (2–5%) of ECs are associated with LS, a cancer predisposition syndrome, a considerably larger proportion, approximately 30%, of universally screened tumors exhibit MMR-d [20]. While IHC loss of MSH2, MSH6, or isolated loss of PMS2 is highly associated with LS and points to a specific MMR gene for subsequent genomic sequencing, MLH1 loss is largely secondary to sporadic *MLH1* CpG island promoter hypermethylation (*MLH1*-PHM), causing transcriptional silencing [21]. Recently, rare cases of LS-like MLH1-deficient (MLH1-d) cancers associated with high-risk constitutional *MLH1* methylation (epimutation) have been described, with a prevalence of <1% of MLH1-d methylated tumors [22]. Therefore, in the context of LS screening, it has been recommended that if MLH1 protein loss is noted on IHC, *MLH1*-PHM testing is reflexively performed to differentiate between sporadic and hereditary MLH1 loss [6,23]. Germline *MLH1* defects are rare in EC and the likelihood of a concurrent germline mutation and hypermethylation is generally thought to be relatively low [24]. Therefore, the presence of *MLH1*-PHM is considered a negative predictor of LS, limiting the need for consultations in cancer genetic clinics and germline analysis to exclude LS. As MLH1-d is detected in over 20% of unselected EC samples, reflex *MLH1*-PHM testing generates substantial costs and burdens on pathology laboratories, partly due to limited assay availability [24].

This study was designed to critically evaluate reflex *MLH1*-PHM testing strategy’s impact, determine the proportion of MLH1-d EC with PHM and assess the associated costs within the publicly funded Canadian healthcare system. We are raising a provocative question: can we assume that the majority of the MLH1-d ECs are due to PHM and forgo further testing in healthcare systems with finite resources? The data could be used to explore opportunities for process improvement, aiming to optimize its effectiveness in identifying individuals with LS, and constitutional hypermethylation syndromes versus those with sporadic MLH1 loss.

## 2. Materials and Methods

### 2.1. Study Population

This retrospective study was approved by the Sunnybrook Health Sciences Centre research ethics board (SUN-5582) and written informed consent was waived. The study included a universally screened population of patients diagnosed with EC at a tertiary-care hospital (Sunnybrook Health Sciences, June 2017–July 2022, *n* = 1295) and a major community laboratory in Ontario, Canada (LifeLabs, January 2018–December 2021, *n* = 1215). A query of the Sunnybrook and LifeLabs Pathology Department laboratory information system was conducted by searching for all EC specimens tested by IHC for MMR protein (MLH1, MSH2, MSH6, and PMS2) expression status followed by an *MLH1*-PHM test when appropriate. Pathology reports for 2510 EC specimens (including curettages, biopsies and hysterectomies), unselected for age, were retrieved. The following information was extracted from the pathology reports for all patients: age at diagnosis, date of primary diagnosis, histological classification and tumor grade, results of MMR IHC, ER-IHC, p53-IHC and *POLE*-mutation status (when available). For patients who had absent MLH1 expression, *MLH1*-PHM results were accessed and recorded.

### 2.2. Universal Tumor Screening Protocol

According to the universal LS screening protocol at the two laboratories, each EC is tested at the time of first available specimen using IHC to detect the expression of the four MMR proteins: MLH1, MSH2, MSH6 or PMS2. If loss of MLH1 is detected, reflex *MLH1*-PHM testing is performed. The presence of *MLH1*-PHM indicates likely non-inherited (sporadic) tumor development and germline genetic testing for LS is not indicated. Individuals with detected MLH1 IHC loss and absence of *MLH1*-PHM were eligible for germline genetic testing and genetic counselling. Consultations at the cancer genetic clinic were recorded, when available, for MLH1-d without PMH cases at Sunnybrook Health Sciences Centre.

### 2.3. Statistical Analysis

Statistical analysis was performed in SPSS 24.0 (IBM Corporation, Armonk, NY, USA). Descriptive statistics were summarized by percentages for categorical variables and by mean/median and range for continuous variables.

### 2.4. Cost Analysis

In this study, *MLH1*-PHM assay was not available on sites and cases were tested at a central laboratory. Incremental burden included block retrieval, office handling for send out, transportation to an outside molecular pathology laboratory, molecular report uploading to the electronic medical record system, follow-up by pathologist, addendum to the original pathology report and follow-up by oncologist. When PHM is not detected, referral to the cancer genetic clinic was suggested.

## 3. Results

### 3.1. Cohort of Universally Screened Endometrial Carcinomas

**Figure 1:**
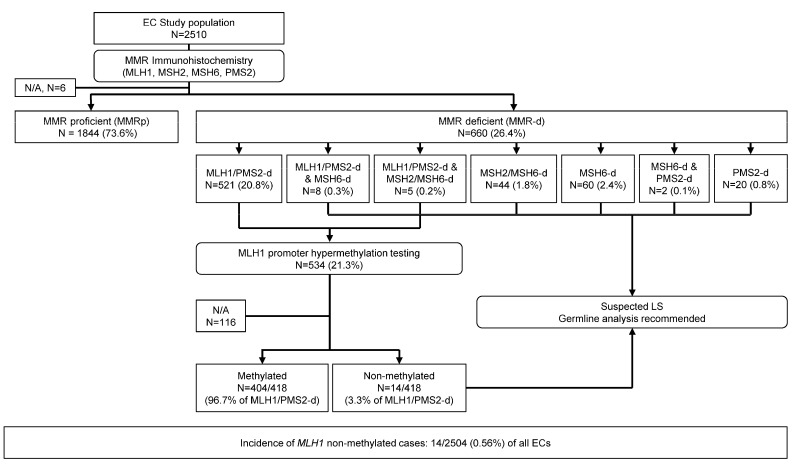
Study Cohort diagram. This study cohort consisted of 2510 consecutive endometrial cancer samples tested by MMR IHC, classified as MMR-proficient and MMR-deficient. MLH1/PMS2-deficient endometrial cancers underwent *MLH1* hypermethylation testing, classified as methylated and not methylated. Patients with loss of MSH2 and/or MSH6, isolated loss of PMS2 and non-methylated MLH1-deficient endometrial cancers were recommended to pursue germline testing for Lynch syndrome.

The study cohort consisted of 2510 consecutive EC samples tested using MMR IHC, classified as MMR proficient (MMRp, *n* = 1844, 73.6%) and MMR-d (*n* = 660, 26.4%, Figure 1). In six cases, MMR protein status could not be determined, and these were excluded from further analysis. Among the MMR-d cases, MLH1/PMS2 loss was detected in 78.9% (521/660) of cases, dual loss of MLH1/PMS2 and MSH6 in 1.2% (8/660) and dual loss of MLH1/PMS2 and MSH2/MSH6 in 0.8% (5/660) of cases. Furthermore, MSH6 loss was identified in 9.1% (60/660) of cases, MSH2/MSH6 loss in 6.7% (44/660) of cases, isolated PMS2 loss in 3.0% (20/660) of cases and MSH6 and PMS2 loss in 0.3% (2/660) of cases.

### 3.2. MLH1 Promoter Hypermethylation Testing Results

MLH1 (and PMS2) loss was detected in 534 cases (21.3%) that were candidates for reflex *MLH1*-PHM analysis. Testing could not be completed due to insufficient material in two cases. Further, testing results were inconclusive in three cases, could not be determined in five cases and were not available in 105 cases. Among these, 44 cases from a major community laboratory were not sent for *MLH1*-PHM testing as it was not routinely performed prior to June 2020. Additionally, 21 tertiary-care hospital cases were consults and their materials were returned following MMR IHC. In 18 *MLH1*-PHM testing was not conducted, but LS testing was undertaken. For 15 cases, *MLH1*-PHM testing was not ordered, while in four cases, it was ordered, but the addendum pathology report was unavailable. In two cases, material was sent to another institution for a clinical trial following MMR IHC, and in one case, *MLH1*-PHM testing was not ordered due to the patient’s age (85). These were excluded from further analysis (Figure 1). Of 418 cases with available testing results, in 404 (96.7%) *MLH1*-PHM was detected and in 14 (3.3%) it was not. Therefore, the incidence of *MLH1* non-methylated cases in our cohort was 14/2504 (0.56%) of all ECs.

### 3.3. Clinicopathologic Features of MLH1-Methylated and Non-Methylated Endometrial Carcinomas

Clinicopathologic features from all 418 cases with available *MLH1*-PHM testing results are summarized in Table 1. The median age of the 404 patients with confirmed *MLH1* hypermethylation was 64 years (range, 39–94). The median age of the 13 non-methylated cases was significantly younger at 56 years (range, 37–71), *p* = 0.018.

*MLH1* methylated cases consisted predominantly of endometrioid tumors (323/404, 80.0%), followed by high-grade ECs (16/404, 4.0%), mixed histology ECs (11/404, 2.7%), dedifferentiated/undifferentiated carcinomas (7/404, 1.7%), carcinosarcomas (6/404, 1.5%), mucinous (3/404, 0.7%) and clear cell (2/404, 0.5%).

Non-methylated cases were also primarily endometrioid tumors (12/14, 85.7%), one (7.1%) dedifferentiated/undifferentiated carcinoma and one (7.1%) serous carcinoma. Statistical analysis revealed no significant differences, although its power was limited by the low number of non-methylated cases. *MLH1* mutational analysis was available for three non-methylated cases, all found to harbor a germline mutation of *MLH1*. Clinical follow-up data as well as any personal or family cancer history data were not available for our cohort.

### 3.4. Cost Analysis of Reflex MLH1 Methylation Testing

The diagnostic process for MLH1-d cases in our cohort involved reflex *MLH1*-PHM analysis in 534 cases (21.3% of cohort). This diagnostic algorithm incites a process utilizing time, labor, and resources of the pathology laboratory.

Cost analysis estimated the expense of office handling, transportation, *MLH1*-PHM testing and molecular reporting to be CAD 231.90 per case, resulting in a total expenditure of CAD 123,834.60 for the 534 cases. Analysis of our cohort revealed that 30 cases need to be tested for *MLH1*-PHM for each additional candidate for *MLH1* germline analysis, resulting in a cost of CAD 6957.00 per candidate.

## 4. Discussion

In this study, we analyzed 2504 EC samples tested with MMR IHC and identified 660 MMR protein deficient cases, with 534 of those showing MLH1/PMS2 loss (21.3%). Cost analysis estimated the expense of *MLH1*-PHM testing to be CAD 231.90 per case, totaling CAD 123,834.60 for the 534 cases, along with significant time and labor burden for the pathology laboratory. *MLH1* non-methylated cases comprised 3.3% (14/418) of MLH1/PMS2-d cases and 0.56% (14/2504) of all cases; this was consistent with prior studies indicating DNA methylation accounts for the vast majority of MLH1/PMS2-d ECs [24]. MMR testing in EC is essential for molecular subtype classification and for LS screening. However, for molecular classification and selection of immunotherapy, current guidelines do not distinguish between MLH1-d with or without PHM; although, some future and ongoing clinical trials are planned to have a subgroup analysis to study its potential effect on response to treatment [15].

Given the rarity of germline *MLH1* defects in EC, a careful scrutiny of reflexive *MLH1*-PHM testing in all MLH1/PMS2-d EC cases is warranted. The current practice entails methylation testing in a substantial number (20–30%) of ECs, leading to notable financial and resource implications [25]. However, the benefits of methylation testing are applicable to a limited number of EC patients, highlighting the importance of assessing resource utilization efficiency and exploring strategies to enhance the cost-effectiveness of *MLH1*-PHM testing. The identification of even a single MLH1-associated LS EC holds great importance, as it facilitates early detection, prevention of other cancer types and extends benefits to family members. Therefore, an optimized approach should be adopted incorporating clinical correlation, family history and judicious consideration of methylation testing to ensure its provision exclusively to those who stand to derive benefit from it.

An additional crucial aspect of current practice that requires scrutiny is how clinicians navigate and analyze *MLH1*-PHM test results. By conducting reflexive *MLH1*-PHM testing on all *MLH1*-d ECs, a substantial number of cases with *MLH1* hypermethylation can be identified. Consequently, given the prevalence of promoter hypermethylation in MLH1-d cases, clinicians may develop an implicitly overlearned association between MLH1-d and *MLH1* promoter methylation, leading them to assume MLH1-d is a sporadic loss [26]. Further, clinicians can initiate treatment without waiting for *MLH1*-PHM results since pembrolizumab has been approved for the treatment of any MMR-d solid tumor, and dostarlimab for MMR-d ECs that have progressed on or after prior therapy, irrespective of the underlying cause being germline or sporadic [27,28,29].

The current *MLH1*-PHM testing approach relies on *MLH1*-hypermethylation as a “hard-stop” in the LS work-up, which can obscure underlying *PMS2* mutations and/or *MLH1* epimutations [22,30]. *PMS2* pathogenic variants underlie a distinct subset of LS-associated ECs that can be masked by *MLH1* hypermethylation due to the current LS screening approach that associates the simultaneous loss of MLH1 and PMS2 with MLH1-d and reflex testing for *MLH1*-PHM [30]. Additionally, the *MLH1*-PHM testing approach overlooks the significance of *MLH1* epimutations, an alternative mechanism for MLH1-d in EC [22,31]. Constitutional *MLH1* epimutation carriers have a predisposition to an early-onset and/or multiple cancers resembling the MLH1-d LS-associated EC phenotype. The exact prevalence of constitutional *MLH1* epimutation in MMR-d ECs is uncertain. Previous studies, primarily focused on colorectal cancer, reported a prevalence of 3–9% among patients meeting the revised Bethesda Guidelines for MSI testing with an MLH1-d tumor, and negative germline genetic test [22,32]. Including tumor *MLH1* methylation as part of the selection criteria slightly increased these frequencies to 3.5–15.6% [22]. A single study of EC reported a frequency of 0.49% (1/206) for constitutional *MLH1* methylation in a hospital-based unselected, consecutive series of EC cases in Japan [33,34]. This underscores the need for the incorporation of clinical correlation and the consideration of germline testing or *MLH1*-PHM testing if the clinical scenario is sufficiently concerning. This prompts further a questioning of the existing threshold for overlooking *PMS2* mutations and *MLH1* epimutations while performing *MLH1*-PHM testing on all MLH1-d ECs to ensure accurate detection of LS-associated EC as well as the judicious use of germline and *MLH1*-PHM testing. Another option is to explore a less technically demanding and more cost-effective assay than *MLH1*-PHM testing, such as EMP2AIP1 IHC, which exhibits high concordance with *MLH1*-PHM (95% concordance, 94.5% sensitivity, 98.1% positive predictive value), showing promise as a surrogate marker [35].

Another aspect to consider when EC premenopausal patients are triaged out of LS screening based on *MLH1*-PHM or not tested for *MLH1* epimutations are the implications on fertility and ovarian preservation approaches to therapy. According to the NCCN guidelines ovarian preservation can be considered in selected low risk EC patients without LS [11]. Of the 534 patients with MLH1-d tumors in our audit, there were three aged 34–39 and sixteen aged 40–49 to whom this consideration would apply.

The study’s limitations lie in its retrospective design and being confined to two institutions. Nevertheless, our findings provide valuable insights into real-world outcomes of *MLH1* PHM testing, aiding the identification of an optimal screening strategy for clinical practice.

## 5. Conclusions

This study analyzed a large series of universally screened ECs and found that MLH1/PMS2 loss due to hypermethylation is the predominant cause of MMR-d. Therefore, the cost and significant resource implications of reflexive *MLH1*-PHM testing in all MLH1/PMS2-d ECs need to be carefully evaluated. Clinicians should consider the limited benefits of methylation testing in the majority of MLH1/PMS2-d ECs and consider adopting an optimized approach that incorporates clinical correlation, family history and judicious use of methylation testing to ensure it is provided only to those who would benefit.

## Figures and Tables

**Table 1:** MLH1-deficient cases with available *MLH1* promoter methylation analysis results.

	*MLH1* Non-Methylated *n* = 14	*MLH1* Methylated *n* = 404	*p*-Value
Age, median (range)	57 (37–71)	64 (39–94)	0.018
Histological type			0.686
Endometrioid	12 (85.7%)	323 (80.0%)	
G1	6	137	
G2	5	141	
G3	1	44	
N/A	-	1	
Mucinous	-	3 (0.7%)	
Clear cell	-	2 (0.5%)	
Carcinosarcoma	-	6 (1.5%)	
Mixed	-	11 (2.7%)	
Serous	1 (7.1%)	-	
Dedifferentiated/Undifferentiated	1 (7.1%)	7 (1.7%)	
High grade carcinoma, unclassifiable	-	16 (4.0%)	
N/A	-	36	
P53			0.051
Wild-type	4 (66.7%)	168 (94.4%)	
Abnormal	2 (33.3%)	10 (5.6%)	
N/A	8	226	
ER			0.492
Positive	3 (21.4%)	69 (85.2%)	
Negative	1 (7.1%)	12 (14.8%)	
N/A	10	323	
*POLE*			-
Wild-type	-	9 (2.2%)	
Mutated	-	-	
N/A	13	395	

## Data Availability

The data that support the findings of this study are available on request from the corresponding author, A.P.
